# State Registration

**Published:** 1913-05-03

**Authors:** 


### State Registration.

DEPUTATION TO THE PRIME MINISTER.

Under the auspices of the Central Committee for State Registration of Trained Nurses a deputation representing a number of organisations waited upon the Prime Minister in his private room at the House of Commons on Monday for the purpose of seeking the support of the Government towards the scheme for the State registration of nurses.

Dr. Chapple, M.P., who introduced the deputation, stated that during recent months there has been more unanimity amongst all those engaged in the nursing profession than there has ever been before, and they were hopeful, if Government facilities were given, that the proposed measure would have an easier passage through the House than it could possibly have had previously.

Mr. Asquith, in replying, stated he agreed that the question was of growing importance, and that our nursing system should be recruited from the best possible sources, and should be carried on by persons who are thoroughly qualified to do such responsible work. I understand it to be your view, he added, that by the machinery which this Bill proposes to set up, at any rate in regard to two of its objects—I do not suppose anyone would contest the third—you obtain as regards qualities or qualifications better security than you have at present. I am far from saying I am not impressed; I am very much impressed with the arguments which you have brought forward, and I recognise, as no one could fail to, the representative and authoritative character, as Sir Victor Horsley very properly said—and he claims to speak as the mouthpiece—of the number of organisations which have publicly expressed a representative opinion on the subject. On the other hand, I think Dr. Chapple takes a very much too sanguine view that the opposition to the proposals has diminished, either in amount or in authority. I won't go into details, but I am informed on the best authority that those who are opposed to the change are ninety-one chairmen of hospitals, most of whom have attained their position as chairmen not by nomination but by free election on the part of subscribers. There are sixty-six matrons in London and 178 matrons in provincial hospitals also opposed to the proposals. He could not honestly say that the Government could give facilities of its time until the somewhat formidable opposition had been substantially mitigated.

[A full account of the proceedings of the deputation is contained in *The Nursing Mirror* this week.]

